# Cost‐effectiveness of routine viral load monitoring in low‐ and middle‐income countries: a systematic review

**DOI:** 10.1002/jia2.25006

**Published:** 2017-11-24

**Authors:** Ruanne V Barnabas, Paul Revill, Nicholas Tan, Andrew Phillips

**Affiliations:** ^1^ Global Health, Medicine, and Epidemiology University of Washington Seattle WA USA; ^2^ Center for Health Economics University of York York United Kingdom; ^3^ Infection and Population Health University College London London United Kingdom

**Keywords:** HIV viral load, cost, cost‐effectiveness

## Abstract

**Introduction:**

Routine viral load monitoring for HIV‐1 management of persons on antiretroviral therapy (ART) has been recommended by the World Health Organization (WHO) to identify treatment failure. However, viral load testing represents a substantial cost in resource constrained health care systems. The central challenge is whether and how viral load monitoring may be delivered such that it maximizes health gains across the population for the costs incurred. We hypothesized that key features of program design and delivery costs drive the cost‐effectiveness of viral load monitoring within programs.

**Methods:**

We conducted a systematic review of studies on the cost‐effectiveness of viral load monitoring in low‐ and middle‐income countries (LMICs). We followed the Cochrane Collaboration guidelines and the PRISMA reporting guidelines.

**Results and Discussion:**

We identified 18 studies that evaluated the cost‐effectiveness of viral load monitoring in HIV treatment programs. Overall, we identified three key factors that make it more likely for viral load monitoring to be cost‐effective: 1) Use of effective, lower cost approaches to viral load monitoring (e.g. use of dried blood spots); 2) Ensuring the pathway to health improvement is established and that viral load results are acted upon; and 3) Viral load results are used to simplify HIV care in patients with viral suppression (i.e. differentiated care, with fewer clinic visits and longer prescriptions). Within the context of differentiated care, viral load monitoring has the potential to double the health gains and be cost saving compared to the current standard (CD4 monitoring).

**Conclusions:**

The cost‐effectiveness of viral load monitoring critically depends on how it is delivered and the program context. Viral load monitoring as part of differentiated HIV care is likely to be cost‐effective. Viral load monitoring in differentiated care programs provides evidence that reduced clinical engagement, where appropriate, is not impacting health outcomes. Introducing viral load monitoring without differentiated care is unlikely to be cost‐effective in most settings and results in lost opportunity for health gains through alternative uses of limited resources. As countries scale up differentiated care programs, data on viral suppression outcomes and costs should be collected to evaluate the on‐going cost‐effectiveness of viral load monitoring as utilized in practice.

## 1 Introduction

Routine viral load monitoring for HIV‐1 management of persons on antiretroviral therapy (ART) has been recommended by the World Health Organization (WHO) since 2013 as the preferred method to identify treatment failure 1. In the initial WHO global HIV treatment guidelines (2003), viral load monitoring was not recommended in low‐ and middle‐income countries (LMICs) due to the requirements for transportation of plasma specimens under controlled conditions, limited laboratory infrastructure and availability of the assay, and relatively high cost of viral load monitoring. Instead, clinical monitoring and/or CD4 count measurement were used to detect treatment failure 2. Over the next ten years the availability of viral load testing increased, the cost of viral load assays decreased, concerns over resistance accumulation at an individual and population level grew, methods for viral load testing on dried blood spot (DBS) specimens were developed, ART guidelines changed to recommend ART for all HIV‐positive persons, and the cost of first‐ and second‐line ART decreased 3. These changes challenged the initial recommendation not to use viral load to monitor the outcome of HIV treatment. However, viral load testing represents a substantial cost in resource constrained health care systems in LMICs.

HIV policymakers and program managers are concerned with affordability and costs because the implications of costs affect the health care they can provide from their limited available resources (i.e. costs imply lost opportunities for generating health) 4. Cost‐effectiveness analysis is a widely‐applied approach to guide whether the health benefits of an intervention are large enough compared to its costs, such that its provision from within limited health care resources would represent “value for money.” This essentially requires determining a cost‐per‐unit of health gained (e.g. per quality‐adjusted life year (QALY)‐gained; or disability‐adjusted life year (DALY)‐averted) from alternative ways of providing viral load monitoring in comparison to other approaches (i.e. clinical, routine CD4 monitoring) and, critically, assessing whether the estimated cost per unit of health gain represents value against a benchmark.

Several published studies have concluded that routine viral load monitoring is cost‐effective in LMICs by referencing the cost per DALY to a benchmark of one to three times gross domestic product (GDP) per capita of a country 5, 6, 7. However, given the level of resource constraints in countries, such benchmarks are now widely recognized as being inappropriate to inform value for money assessments and risk lowering population health through diverting investments away from greater priorities 8, 9.

Suitable benchmarks are context‐specific and can be difficult to ascertain. An emerging stream of research has shown that, for general health care, values are much lower than previous acknowledged – for example ~$60 to 100 in Malawi – due to high levels of unmet health needs 10, 11. However, HIV interventions remain overwhelmingly reliant on overseas aid which is specifically ear‐marked for this condition, and the overall level of such aid means in practice that HIV‐related interventions may be cost‐effective at higher values than this (e.g. $300 to $500) 12, 13, 14. A number of papers sought to specifically assess whether viral load monitoring would improve population health compared to committing the required resources to continued scale‐up of ART and have often found that it would not 15, 16. The central challenge then is whether and how viral load monitoring may be delivered such that it would justifiably constitute a component of HIV care, even in the context of the higher cost effectiveness threshold operating for HIV interventions, and does not divert resources away from other priority health care activities that could generate greater health benefits.

We conducted a review of recent studies on the cost‐effectiveness of viral load monitoring in LMICs. Our hypothesis was that key assumptions about program design and costs of monitoring drive the cost‐effectiveness of programs that incorporate viral load monitoring. The primary review objective was to identify characteristics of programs that make viral load testing more or less likely to be cost‐effective and contribute to improvements in population health, recognizing there are other calls on limited HIV program resources.

## 2 Methods

We reviewed cost and cost‐effectiveness studies of viral load testing in HIV treatment programs in LMICs and summarized the key factors that determine cost‐effectiveness. We followed Cochrane Collaboration guidelines in conducting our review 17, and PRISMA guidelines in reporting results 18.

### 2.1 Search strategy, selection of data, synthesis

We conducted an electronic search of the PubMed database on 14 February 2017 for studies published from 1 January 2000, onwards, using the following MeSH terms: (“viral load”(MeSH Terms) OR “viral”(All Fields) AND “load”(All Fields) OR “viral load”(All Fields)) AND (“economics”(Subheading) OR “economics”(All Fields) OR “cost”(All Fields) OR “costs and cost analysis”(MeSH Terms) OR (“costs”(All Fields) AND “cost”(All Fields) AND “analysis”(All Fields)) OR “costs and cost analysis”(All Fields)). The EMBASE data base was searched using the equivalent search terms. Reference lists of papers meeting criteria were hand searched for additional articles. To ensure that we included unpublished data, abstracts were reviewed from the past meetings of the Conference on Retroviruses and Opportunistic Infections (CROI).

Abstracts and full‐text articles of potentially relevant studies were reviewed independently by two authors (N.T. and R.V.B.) against pre‐defined criteria. Papers were eligible for inclusion if the analysis was conducted in a LMIC setting and a cost‐effectiveness result for viral load monitoring was included. Data were extracted using a standardized data extraction form. Discrepancies regarding eligibility of papers were discussed and consensus reached. The methodological quality of included studies was reviewed by N.T. and R.V.B. Discrepancies in quality rating were discussed and consensus reached. Studies were rated as low, moderate, or high risk of bias, dependent on whether they met standard guidelines for health economic evaluation reporting 19. The study results were summarized and synthesized for discussion; a quantitative summary statistic was not estimated for the cost‐effectiveness of viral load monitoring. To assess factors that increased the cost‐effectiveness of viral load monitoring, we reviewed the model and program parameters that determined cost‐effectiveness relative to the base case and qualitatively summarized and grouped the factors into three main themes.

## 3 Results

The electronic search yielded 1248 results of which 1165 were unique abstracts. We identified 23 manuscripts and four conference abstracts for review of which 18 met the search criteria and addressed the cost‐effectiveness of viral load monitoring (Table 1).

**Table 1:** Studies estimating the cost‐effectiveness of viral load (VL) monitoring

First author	Location	Year studied	Key features modelled	Authors’ conclusion on cost‐effectiveness+	Factors that make VL testing more cost‐effective	Incremental cost‐effectiveness ratio (ICER)
Kahn 21	Tororo, Uganda	2001 to 2002	Randomized trial	Definite no	VL testing was not effective in this RCT (no evidence of health benefit)	Clinical + CD4 + VL monitoring vs. Clinical + CD4 monitoring: $5181 per DALY averted
Schneider 31	Thailand	2001 to 2009	Heterogeneity in VL Virologic failure Effect of VL on differentiation of care	^Qualified yes	Lower cost of ART	Annual monitoring after single screen at six months vs. Supplying only first‐line ART for ten years (with ART costs): $68,084 per QALY Annual monitoring after single screen at six months vs. Supplying only first‐line ART for ten years (without ART costs): $7224 per QALY
Bishai 22	Sub‐Saharan Africa	2004 (Cost data)	Heterogeneity in VL Virologic failure	^Qualified yes	Second line treatment would have to be available Cost of VL testing would have to be reduced to $14 per test to have same median ICER as CD4 testing compared to clinical monitoring	VL vs. CD4 monitoring (second line unavailable): $16,139 per QALY VL vs. CD4 monitoring (second line available): $14,670 per QALY
Vijayaraghavan 32	South Africa	2005	Heterogeneity in VL Virologic failure Variation switching to second line in first line failures	Definite yes	Treating patients with VLs >100,000 copies/ml to reduce HIV transmission by “highly efficient transmitters”	Use of VL testing every six months vs. WHO guidelines: $7860 per QALY Increased VL testing frequency (every three months) vs. WHO guidelines: $41,286 per QALY
Pham 30	Vietnam	2005 to 2013	Resistance Virologic failure	^Qualified yes	VL testing every two years and individuals with VL >1000 copies/ml and detectable HIV drug resistance placed on second line ART Lower cost of second line ART Low cost POC‐VL and resistance tests	WHO recommendations for VL monitoring (six months after treatment initiation and every 12 months thereafter) vs. Status quo (no VL monitoring): $5243 per DALY averted
Kimmel 27	Côte d'Ivoire	2006	Heterogeneity in VL Resistance	^Qualified yes	HIV RNA test <$90 Decrease in second line efficacy due to time spent on failing first line ART is greater than 1% per month Cost of second line ART <$300	VL monitoring (test cost = $50 to $87) vs. Cotrimoxazole prophylaxis for opportunistic infections: $1990 to 2920 per YLS
Boyer 20	Cameroon	2006 to 2010	Clinical Trial	Qualified no	Lower priced generic in‐house assay Use VL assay for patients with CD4 <200 cells/μl	Clinical monitoring vs. VL + CD4 + clinical monitoring (Abbot RealTime HIV‐1 assay): $4768 per LYS Clinical monitoring vs. VL + CD4 + clinical monitoring (Generic assay): $3339 per LYS
Bendavid 24	Cape Town area	2007	Heterogeneity in VL Virologic failure	^Qualified yes	Lower price of VL testing Possibly reduced HIV transmission (not modelled) Fewer accumulated resistance mutations (not modelled) Higher rate of virologic failure	VL monitoring + CD4 vs. CD4: $5414 per LYG Every three months vs. every six months: $100,000 per LYG
Phillips 34	Lower to middle income countries	2008	Heterogeneity in VL Virologic failure Resistance Variation in switching to second line in first line failures	Qualified no	Lower cost of second line ART	VL >500 copies/ml vs. Switch after WHO stage four event: $1500 per LYG VL >10,000 copies/ml vs. Switch after WHO stage four event: $4011 per LYG
Scott Braithwaite 16	Sub‐Saharan Africa	2008 (Cost data)	Virologic failure Heterogeneity in VL	Qualified no	ICER for VL testing better when first and second line costs are equal Use routine virological testing when ART is already initiated at 500 cells/μl and coverage targets have been met Low cost VL testing Six monthly VL testing, switching threshold at 1000 copies/ml is the only strategy on the efficient frontier	VL monitoring (10,000 copies/ml to 1000 copies/ml threshold) vs. Starting ART at CD4 count 200 cells/μl: $4723 to $25,370 per QALY
Estill 5	LMIC (Cost data can be updated for specific setting)	2010	Variation in switching to second line in first line failures Resistance Effect of VL on differentiated care Virologic failure Heterogeneity in VL	Qualified no	Routine VL monitoring cost‐effectiveness depends on cost of second line ART POC VL cost‐effectiveness improved if first and second line ART prices are close Targeted VL monitoring is cost‐efficient only if second line costs are much higher than first line, and routine VL monitoring does not prevent failure	VL monitoring vs. Clinical monitoring: $951 to $5813 per DALY averted POC‐VL (every six to twenty‐four months) vs. CD4 monitoring (irregular every six months, every six to twenty‐four months): $426 to $33,515 per DALY averted Lab‐VL (every six to twenty‐four months) vs. CD4 monitoring (irregular every six months, every six to twenty‐four months): $984 to $8862 per DALY averted
Hamers 26	South Africa	2011	Virological failure	Definite yes	Reduced accumulation of drug‐resistance mutations, reduced incidence of opportunistic infections and mortality, increased economic productivity, reduced HIV transmission	VL‐only every six months vs. Symptom‐based approach: $3183 per LYG VL‐only every 12 months vs. Symptom‐based approach: $5319 per LYG
Estill 25	LMIC (Cost data can be updated for specific setting)	2012	Heterogeneity in VL Virologic failure	^Qualified yes	Include reductions in HIV transmission with suppression Lower cost of second line ART and VL Risk of virological failure with monitoring strategy (reduced by VL monitoring compared to clinical/CD4 monitoring) Use POC‐VL test level of detection criteria of 1000 copies/ml to reduce unnecessary switches to second line ART	More accurate detection of treatment failure and faster, more appropriate switching to second line: $4010 to $9230 per QALY vs. clinical monitoring and $5960 to $25,540 vs. CD4 monitoring Taking transmission into account + More accurate detection of treatment failure and faster, more appropriate switching to second line: $2450 to $5830 per QALY vs. clinical monitoring and $2230 to $10,380 vs. CD4 monitoring Risk of virologic failure twice as high with clinical or CD4 compared to VL monitoring + Taking transmission into account + More accurate detection of treatment failure and faster, more appropriate switching to second line: $960 to $2500 per QALY vs. clinical monitoring and cost saving $2460 per QALY vs. CD4 monitoring
Keebler 15	Zambia	2012	Presents results from three different models Heterogeneity in VL Resistance – HIV Synthesis model (Phillips), Braithwaite and colleagues Variation in switching to second line in first line failures Virological failure	Qualified no	VL monitoring after high ART coverage is achieved Lower second line ART cost Lower test costs Targeted VL strategy	*VL every 12 months vs. VL every 36 months:* Braithwaite (20 years): $6018.83 per DALY averted HIV Synthesis (15 years): $3413.8 per DALY averted Estill (five years): $3760 per DALY averted
Negoescu 28	Uganda	2013	Virologic failure Effect of VL on differentiated care	^Qualified yes	Client centered and tailored to country GDP: Adjusting VL monitoring intervals of HIV patients on ART according to individual patient characteristics, disease dynamics, behavior, and GDP. Implementation in high resource settings	Adaptive VL optimized to 1× GDP threshold vs. monitoring every 24 months: $491 per QALY Adaptive VL optimized to 3× GDP threshold vs. adaptive VL optimized to 1× GDP threshold: $1311 per QALY
Ouattarra 29	Côte d'Ivoire	2013 to 2017	Virologic failure Effect of VL on differentiated care Heterogeneity in VL	Definite yes	Adaptive VL ICER <1× GDP if second line ART and VL costs decreased to $156 and $13 Sensitive to initial CD4 count of cohort Lower HIV transmission rate due to monitoring (not modelled)	Adaptive VL vs. VL confirmation: $4100/YLS (2013 USD)
Phillips 35	Zimbabwe	2015 to 2025	Paper was primarily focused on whether use of drug resistance testing was likely to be cost effective as part of ART monitoring strategy. Variation in switching to second line in first line failures Virologic failure Resistance Heterogeneity in VL	Qualified no	Most effective strategy for DALYs averted was VL monitoring without confirmation	VL monitoring with no confirmation vs. no monitoring, no second line: $2113 per DALY averted
Phillips 23	Zimbabwe	2015 to 2035	Variation in switching to second line in first line failures Virologic failure Resistance Heterogeneity in VL Effect of VL on differentiated care	^Qualified yes	With $22 viral‐load test cost, annual savings of $30 needed to make program cost‐effective. Reducing visits from every one to three months to every six months or every nine to twelve months should enable these savings. Reduction in non‐ART program costs Use VL monitoring less frequently than every 12 months (caveat: health risks with such infrequent VL monitoring not well understood)	DBS VL monitoring every 12 months vs. No monitoring: $326 per DALY averted ((if used to differentiate care and reduce clinic visit costs)

^Qualified yes – the authors’ overall conclusion was that viral load monitoring was cost effective, but that this was conditional on the existence of certain conditions, +The author's conclusions on cost effectiveness depend on the choice of cost effectiveness threshold – the appropriate threshold is now recognised as being lower than had previously been supposed, particularly when using the 1× or 3× GDP criteria. ART, antiretroviral therapy; DALY, disability‐adjusted life years; GDP, gross domestic product; LYG, years of life gained; LYS, life years saved; QALY, quality‐adjusted life years; VL, viral load; YLG, years of life gained; WHO, World Health Organization; LMIC, low‐ and middle‐income countries.

The studies were conducted in a range of LMIC settings (sub‐Saharan Africa, Cameroon, Uganda, South Africa, Zambia, Zimbabwe, Cote d'Ivoire, Vietnam and Thailand). Two health economic analyses were based on clinical trials conducted in Uganda and Cameron 20, 21. The remaining analyses collated surveillance data, worked closely with programs, and reviewed the literature to obtain parameters for health economic modeling. Study outcomes were reported as the cost per DALY averted, cost per QALY gained, cost per life year gained (LYG), and/or year of life saved (LYS).

Cost‐effectiveness analyses were conducted from 2004 22 and projected out to 2035 23. Twelve 5, 22, 23, 24, 25, 26, 27, 28, 29, 30, 31, 32 of the 18 studies found that viral load monitoring was cost‐effective. Studies used different thresholds to determine cost‐effectiveness combined with variation in the unit costs resulted in marked heterogeneity between the results. The unit cost of viral load testing varied in the analysis from $5.80 25 to $103.88 27 per test, and the annual cost of first line ART and second line ART varied also from $108.18 5 to $462.47 30 and $239.31 33 to $2071.33 30, respectively (2017 USD). The range in the costs for viral load assays and ART is due to agreements with manufacturers, volume of demand, advocacy, human resource costs, and calendar time of the study (with costs generally falling over time). Studies that did not include the transmission benefits of viral suppression may have underestimated the cost‐effectiveness of viral load monitoring 27, 29. Neither clinical trial found a beneficial health impact of viral load monitoring on clinical outcomes 20, 21, thus the change in costs was not balanced by an improvement in the health outcomes with the intervention. The clinical trials were conducted prior to lower cost viral load testing and ART recommended for all HIV‐positive persons.

## 4 Discussion

We found three main factors that make it more likely for viral load monitoring to be cost‐effective (Table 2): 1) Use of effective, lower cost approaches to viral load monitoring; 2) Ensuring the pathway to health improvement is established and that viral load results are acted upon; and 3) Simplifying HIV care and including viral load monitoring to facilitate differentiated care.

**Table 2:** Characteristics that support cost‐effective viral load (VL) monitoring for HIV: 1) low cost approaches; 2) pathway to impact; and 3) differentiated care

Characteristic	Comment	Ref.
	**Effective, low cost approaches to VL monitoring**	
Lower average unit costs: a VL assays, b Other factors contributing to fully loaded costs for VL monitoring (e.g. personnel, transport, facility costs etc.), c Dried blood spots (DBS) replacing plasma specimens d Second line ART	Roche introduced $9.40 price ceiling for VL testing in 2014 (fully loaded $20). DBS allows more feasible collection and transport for specimens in challenging conditions, at lower cost compared to transportation of plasma specimens. Decreases in the cost of protease inhibitor based second line ART regimens would decrease the cost of changing to second line regimens if clients are failing first line therapy. Decreases in these variable unit costs can drive cost‐effectiveness.	16, 22, 23, 24, 25, 27, 29, 30, 31
Less frequent VL testing	VL monitoring six months after initiation and then bi/annually was cost‐effective compared to six monthly monitoring. Two‐yearly testing vs. annual testing increases cost‐effectiveness	1, 23, 29
	**Pathway to impact: Action based on VL results**	
Action based on VL results	Most models assume that the VL results are acted on in a timely manner with adherence counseling, resistance testing if available, and prompt switch to second line ART which increases viral suppression. Assuming that a high proportion of tests fail or the results are not received dramatically decreases the likelihood of cost‐effectiveness Timely (<six months) switch to second line ART in people with consecutive viral load levels >1000 copies/ml. which minimizes resistance and clinical failure.	23
	**Differentiated care for HIV**	
VL informed differentiated care	Potential to save costs from fewer clinical visits, longer prescriptions, clinical visits with regular clinical staff for clients who achieve viral suppression and do not require complex specialty care.	15, 23, 36, 37, 38, 39

ART, antiretroviral therapy.

### 4.1 Effective, low‐cost approaches to viral load monitoring

Several factors can ensure the cost of viral load testing and the fully loaded cost (all the costs of conducting a test) is as low as possible: choosing an efficient specimen for measuring viral load such as DBS, using an assay and threshold that strikes the right balance between the risks of missing detectable viral load and switching unnecessarily, and limiting the frequency of viral load monitoring 16, 22, 23, 24, 25, 27, 29, 30, 31. In 2014, Roche introduced a ceiling price for PCR laboratory based viral load testing of $9.40 (and a fully loaded cost of $20) which, at a quarter of the previous cost, has increased the cost‐effectiveness of viral load testing 29. Lastly, point‐of‐care viral load testing could well be more cost‐effective if the real‐time response to the results which such testing enables improves clinical care.

In addition to the costs of viral load testing itself, analyses have shown that downstream costs (in particular second line ART) notably affect the cost‐effectiveness of viral load monitoring. Although costs of protease inhibitor based regimens have fallen markedly in recent years (from $600 to $205 per patient year in 2016 40), these are still very high for low resource health care systems – limiting the potential for viral load monitoring to be cost effective. The incremental cost‐effectiveness ratio (ICER) of viral load testing changed from $4100 to <1500/year of life saved when considering a range of lower viral load testing and ART costs in Cote d'Ivoire 29.

Similarly, cost‐effectiveness analyses that account for the decrease in transmission benefits with ART better estimate the full health gains compared to analyses only looking at individual benefits and cost.

### 4.2 Pathways to impact: action based on viral load results

There are inevitably challenges in implementing and acting upon viral load testing and these need to be considered in assessing cost‐effectiveness. Specimens need to be transported in an efficient manner to accredited laboratories, results relayed to clinicians and clients, and the results acted on promptly with adequate access to second line ART. Most analyses that find viral load monitoring to be cost‐effective assume that the viral load results are acted on in a timely manner (either immediately or less than six months). Protocols that delineate the next steps for a client on ART with a detectable viral load for example adherence counseling, viral load retesting three months after adherence counseling, resistance testing (where available) if viral load is still detectable despite adherence, and switching to second line ART in a timely manner, increase clinical effectiveness and decrease the emergence and transmission of resistance mutations. Some clients may require more regular visits and monitoring for complex disease or comorbidities. Notably, even with viral load monitoring, switching to second line ART generally does not occur within the timeframe assumed by most mathematical models of HIV and the proportion of people who are on second line regimens is generally below 5% 23. Phillips and colleagues estimated that even avoiding a three‐month delay through point‐of‐care testing, could increase health benefits by 6% with no additional costs. Also, both randomized studies of the cost‐effectiveness of viral load testing found viral load testing not to be cost‐effective for monitoring 20, 21, a note of caution that implementation must emulate the modelled scenarios to meet the cost‐effectiveness thresholds. The cost‐effectiveness of viral load monitoring hinges on adequate services to deliver clinical benefits.

### 4.3 Differentiated care for HIV

Differentiated care for HIV allows simplifying of protocols for persons well controlled on ART, with client responsive viral load testing (six months after initiation and then annually unless clinically indicated) providing feedback to inform on‐going individual and program effectiveness. This also supports viral load monitoring replacing CD4 count monitoring, spacing appointments, providing longer prescription refills, task shifting, community‐based ART, testing using DBS specimens to simplify specimen transport, and using clinical care resources for complex clinical cases and clients who are not suppressed on ART. In this differentiated care context, viral load monitoring enables greater comfort with less clinical engagement. The cost of viral load testing can be offset by savings in the clinical visit costs. Thus, differentiated care with viral load monitoring can save costs with greater health gains compared to standard of care clinic management (for a simulated model population of Zimbabwe over 20 years, viral load monitoring and differentiated care had a cost saving of $139 million and 580,000 DALYS compared to CD4 monitoring, which represents a doubling of the health gain at more than a third less of the cost 23). It is notable that the distinction between standard of care clinic HIV management and differentiated care is blurring as some, but not all, aspects of differentiated care are incorporated into clinic care such as chronic medication refills without clinic visits available for clients on ART in South Africa. Importantly, for differentiated care, viral load monitoring provides program level evidence on whether reduced clinical engagement impacts individual and public health outcomes.

Cost‐effectiveness studies aim to inform the allocation of limited resources. This requires estimating the *incremental costs* of alternative interventions (e.g. viral load vs. CD4 vs. clinical‐only monitoring) and the *incremental health benefits* (e.g. QALYs‐gained or disability adjusted life years (DALYs)‐averted); then assessing whether the cost‐per‐unit of health improvement represents *sufficient value*, compared to other claims on limited resources. These can all change depending upon how and where viral load monitoring is delivered, so any universal claims to cost‐effectiveness are misguided and it is vital to understand the place of viral load monitoring within HIV programs and how it may facilitate design of programs to improve population health from within the resources available.

The results table (Table 1) illustrates that the ICER needs to be interpreted within the context of the analysis. First, the ICERs per QALY gained or DALY averted, even though each measure is in the same units, are not directly comparable unless the same strategies are compared, that is whether viral load testing is compared with clinical staging and/or CD4 count and underlying programmatic assumptions (indicated in the ICER column). Second, the threshold for what is considered cost‐effective does vary as is illustrated in the interpretation of the results (conclusion column) which reflects the perspective and setting of the analysis.

### 4.4 Looking ahead: likely future programmatic changes that impact viral load cost‐effectiveness

Lastly, notable programmatic changes are likely to impact cost‐effectiveness of viral load testing, specifically changing to ART regimens with a high barrier to resistance 27 such as integrase inhibitors. The integrase inhibitor, dolutegravir, for example, has a higher barrier to resistance than current first line efavirenz based regimens, which could decrease the clinical benefits of viral load monitoring since resistance is encountered less frequently. The combination of ART formulations and monitoring strategy should offer the greatest health gains for the cost. With alternative monitoring tests, for example detecting TDF/TAF in urine, viral load monitoring might only be required for clients without detectable TDF/TAF or other clinical concerns. As new regimens and models for care are rolled out, the cost‐effectiveness of viral load monitoring will need to be reassessed on a continual basis.

## 5 Conclusions

The cost‐effectiveness of viral load monitoring is critically dependent on context. Viral load monitoring in differentiated care programs provides evidence that reduced clinical engagement, where appropriate, is not impacting health outcomes 23. To achieve this goal of cost effective viral load monitoring, differentiated care programs will need to be scaled up to achieve the gains of cost saving – introducing viral load monitoring without differentiated care can result in lost opportunity for health gains through an alternative use of resources. As countries scale up differentiated care programs, data on viral load outcomes and cost are essential to evaluate the on‐going cost‐effectiveness of viral load monitoring in practice. Efforts to standardize this reporting and rapid analysis would facilitate the adoption of successful differentiated care strategies.

## Competing interests

The author have no competing interests.

## Authors’ contributions

RVB, PR, and AP oversaw the review. RVB wrote the first draft of the paper, which was revised by all authors. NT did the electronic searches and reviewed the abstracts, with guidance from RVB. All authors contributed to design and execution of the review, as well as to the interpretation of findings. All the authors approved the final version of the paper for submission.
